# Fibroglandular Tissue and Background Parenchymal Enhancement on Breast MR Imaging Correlates With Breast Cancer

**DOI:** 10.3389/fonc.2021.616716

**Published:** 2021-09-30

**Authors:** Xiaoxin Hu, Luan Jiang, Chao You, Yajia Gu

**Affiliations:** ^1^ Department of Radiology, Fudan University Shanghai Cancer Center, Shanghai, China; ^2^ Department of Oncology, Fudan University Shanghai Medical College, Shanghai, China; ^3^ Center for Advanced Medical Imaging Technology, Shanghai Advanced Research Institute, Chinese Academy of Sciences, Shanghai, China

**Keywords:** BPE, breast, quantitative assessment, breast parenchymal enhancement rate, MRI

## Abstract

**Objectives:**

To evaluate the association of breast cancer with both the background parenchymal enhancement intensity and volume (BPE_I_ and BPE_V_, respectively) and the amount of fibroglandular tissue (FGT) using an automatic quantitative assessment method in breast magnetic resonance imaging (MRI).

**Materials and Methods:**

Among 17,274 women who underwent breast MRI, 132 normal women (control group), 132 women with benign breast lesions (benign group), and 132 women with breast cancer (cancer group) were randomly selected and matched by age and menopausal status. The area under the receiver operating characteristic curve (AUC) was compared in Cancer vs Control and Cancer vs Benign groups to assess the discriminative ability of BPE_I_, BPE_V_ and FGT.

**Results:**

Compared with the control groups, the cancer group showed a significant difference in BPE_V_ with a maximum AUC of 0.715 and 0.684 for patients in premenopausal and postmenopausal subgroup, respectively. And the cancer group showed a significant difference in BPE_V_ with a maximum AUC of 0.622 and 0.633 for patients in premenopausal and postmenopausal subgroup, respectively, when compared with the benign group. FGT showed no significant difference when breast cancer group was compared with normal control and benign lesion group, respectively. Compared with the control groups, BPE_I_ showed a slight difference in the cancer group. Compared with the benign group, no significant difference was seen in cancer group.

**Conclusion:**

Increased BPE_V_ is correlated with a high risk of breast cancer While FGT is not.

## Introduction

The mammographic density of the breast has been found to provide valuable information regarding the risk of breast cancer (1–6). Among 12 single-nucleotide polymorphisms associated with a high risk of breast cancer, at least 3 are associated with changes in the breast density (7–9)., Mammography is usually used to obtain two-dimensional images of the breast, but this technique is inaccurate for evaluation of the breast density, which mainly comprises fibrous and glandular tissue (10). Magnetic resonance imaging (MRI) can be used to accurately evaluate the fibroglandular tissue (FGT) in three dimensions with a dynamic contrast-enhanced technique. The level of background parenchymal enhancement (BPE) reflects the features of enhanced FGT. Some studies have demonstrated that the breast tissue showing enhancement upon imaging is correlated with the risk of breast cancer. In one study, an increased level of BPE was associated with a higher risk of breast cancer (11).

King et al. (12) explored the relationships between breast cancer and both FGT and BPE on MRI. The odds radio for moderate or marked BPE versus minimal or mild BPE was 10.1; when patients with breast cancer were compared with normal controls, this odds ratio was significantly better than that for the breast X-ray density (5, 6). These results indicate that BPE has a stronger association with the risk of breast cancer than FGT, especially when evaluating the epithelial mammary gland blood supply. However, the authors’ assessment of FGT and BPE was subjective and lacked inter-observer consistency (12). Quantitative BPE is objective and accurate, which can provide reproducible data.

Previous published studies have shown that both FGT and BPE are associated with the concentrations of hormones such as estrogen and progesterone. Such associations were shown to be influenced by the patient’s age, menstrual cycle, menopausal status, therapy with aromatase inhibitors or tamoxifen, and hormone replacement therapy (13–18). Therefore, in the present study, patients taking endocrine therapy or hormone replacement therapy were excluded to avoid the influence of these treatments. The patients were then matched for age, menopausal status, and menstrual cycle. BPE is described in terms of both its intensity and volume (BPE_I_ and BPE_V_, respectively). The purpose of this study was to examine the relationship between quantitative FGT/BPE and the risk of breast cancer.

## Materials and Methods

### Patients and Study Design

The institutional review board granted a waiver of authorization and patient consent for our retrospective study, which was in compliance with the Health Insurance Portability and Accountability Act (HIPAA). From January 2009 to December 2013, we conducted a retrospective review of 17,274 consecutive women who underwent breast MRI examinations in our hospital. Of these 17,274 women, 472 had bilaterally normal breasts on MRI (Breast Imaging-Reporting and Data System category 1). They had no lesions on MRI, ultrasound, or mammography examinations in the subsequent 2 years. Among these 472 women, only 132 met the following criteria for enrollment in the control group: Undergoing no hormonal therapy and having a 4-week menstrual cycle. Patients in the control group were matched 1:1 to patients in the breast cancer group by: 1) similar age (within a range of 5 years) 2) consistent menopause status 3) consistent menstrual cycle. Patients in the cancer group had untreated unilateral breast cancer diagnosed by operation or biopsy. Similarly, 132 patients who had unilateral breast benign lesions confirmed by biopsy or operation were enrolled in the benign group. These patients all had unilateral benign breast lesions. Patients in three groups were classified into premenopausal and postmenopausal subgroups according to the menopause status. Based on a 4-week menstrual cycle, we subclassified the premenopausal women into four categories: those in the first, second, third, and fourth week after menstruation.

### MRI Protocol

MRI examinations were performed using a 1.5T breast MRI scanner (Aurora Imaging Technology, Inc., North Andover, MA, USA) with breast coil. We examined the patients’ bilateral breast and axillary areas in the prone position with natural ptosis. We injected gadolinium-diethylenetriamine pentaacetic acid (Gd-DTPA) at 0.2 mmol/kg for dynamic contrast-enhanced MRI examination at a flow rate 2.0 mL/s with a high-pressure syringe. Next, 15 mL of 0.9% sodium chloride solution was injected to flush the remaining Gd-DTPA. The MRI series included one positional reference image, one T1-weighted non-fat-suppressed image, one T2-weighted fat-suppressed image, one pre-contrast T1-weighted fat-suppressed image, and three post-contrast T1-weighted fat-suppressed images. The time phases examined were the early phase (2 min), middle phase (4 min), and late phase (6 min) after contrast injection. The scanning parameters are shown in **Supplementary Table 1**.

### MRI Evaluation

We developed a fully automated scheme for quantitative analysis of FGT and BPE from dynamic contrast-enhanced MRI (19). One pre-contrast T1-weighted fat-suppressed image was used to evaluate FGT and one pre-contrast T1-weighted fat-suppressed image and three post-contrast T1-weighted fat-suppressed images was used to evaluate BPE_V_ and BPE_I_. After contrast agent administration we evaluated BPE_I_ and BPE_V_ at 60, 180, and 300 s (k-space center time).This fully automated method consists of three steps: segmentation of the whole breast, segmentation of FGT, and segmentation of enhanced FGT. Based on the automatically extracted volume of interest, a dynamic programming method was applied in each two-dimensional slice of a three-dimensional MRI scan to delineate the chest wall and breast skin line for segmenting the whole breast. This step took advantage of the continuity of the chest wall and breast skin line across adjacent slices. We then used the fuzzy c-means clustering method with automatic selection of the cluster number for segmenting of the FGT within the segmented whole breast area. Finally, a statistical method was used to establish a threshold based on the estimated noise level for segmenting the enhanced FGT in the subtraction image of the pre- and post-contrast MRI scans.

**Figure 1:**
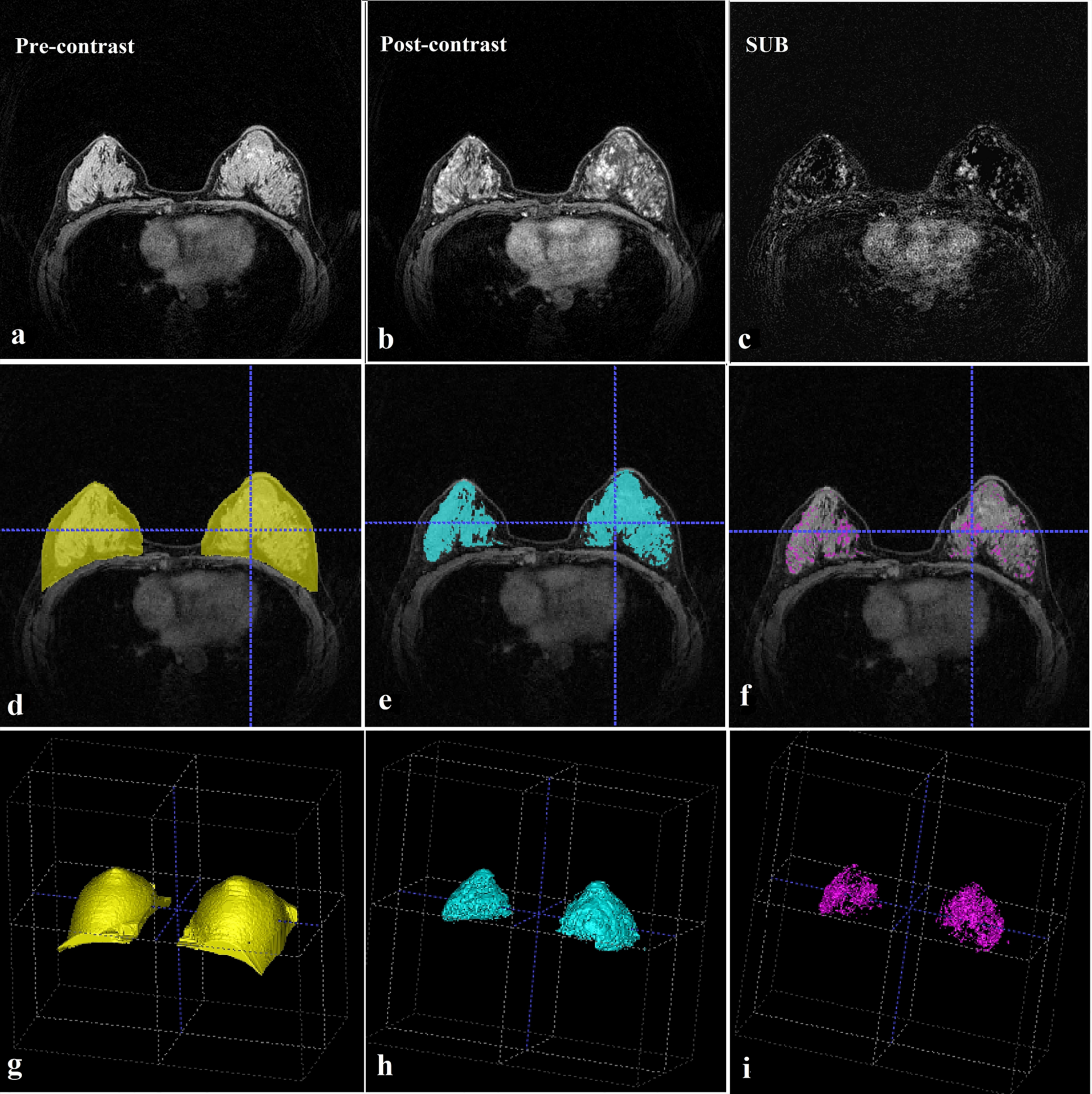
MRI evaluation. **(A)** Pre-contrast breast MRI (T1-weighted fat-suppressed sequence). **(B)** This post-contrast (early) image corresponds to the T1-weighted image shown in **(A)**. **(C)** Subtraction image from **(A, B)**. **(D)** Whole breast segmentation (yellow). **(E)** Fibroglandular tissue segmentation (blue). **(F)** Enhanced fibroglandular tissue segmentation (purple). **(G)** Whole breast segmentation (3D). **(H)** Fibroglandular tissue segmentation (3D). **(I)** Enhanced fibroglandular tissue segmentation (3D). MRI = magnetic resonance imaging, 3D = three-dimensional.

FGT and BPE were quantitatively assessed based on the segmented whole breast, FGT, and enhanced FGT (segmented tissues are shown in **Figure 1**). FGT was calculated using the volume ratio of the segmented FGT and breast tissues, BPE_V_ was calculated using the volume ratio of the segmented enhanced and unenhanced FGT, and BPE_I_ was calculated using the enhanced intensity ratio of the segmented enhanced FGT. The following mathematical formulas were used:


FGT=Vfibroglandular tissues/Vbreast tissuesBPEV=Venhanced fibroglandular tissues/Vfibroglandular tissuesBPEI=∑enhanced fibroglandular tissuesIsubtraction imageIoriginal imageNumenhanced fibroglandular tissues

In these formulas, *I_subtraction image_* equals to the intensity of enhanced FGT in the subtraction image and *I_original image_* equals to the intensity of enhanced FGT in the original image.

To avoid effects induced by the lesion, we chose the mean value between the bilateral breasts as the measurement target in the control group. We also chose the contralateral breast in the benign and cancer groups.

### Statistical Analysis

Patients in this study were divided into three groups: the cancer, benign, and control groups. Mann-Whitney U test was used to compare the differences between the cancer group and the normal control group, and between the cancer group and the benign lesion group. Due to the significant difference between FGT and BPE before and after menopause (13, 15), so we’ve also divided patients into premenopausal and postmenopausal subgroups.

Based on the relative consistency of the background parenchyma in the bilateral breasts, we drew receiver operating characteristic curves of FGT, BPE_I_, and BPE_V_ for both the premenopausal and postmenopausal patients using a nonparametric method. We evaluated the discriminative performance of this association calculating modeling using the area under the receiver operating characteristic curve (AUC). All reported P values are two-sided. All analyses were performed using SPSS 16.0 (SPSS Inc., Chicago, IL).

## Results

### Characteristics of the Study Groups

Among all 396 menstrual cycle-matched patients enrolled in the three groups (**Table 1**). All the included women had not received hormone therapy or radiation therapy. In the cancer group, 111 (84%) patients had infiltrating ductal carcinoma, 16 (12%) had ductal carcinoma in situ, 3 (2%) had infiltrating lobular carcinoma, 1 (1%) had sarcomatoid carcinoma, and 1 (1%) had adenoid cystic carcinoma. In the benign group, 89 (68%) patients had mastopathy, 32 (24%) had fibroadenoma, 8 (6%) had intraductal papilloma, and 3 (2%) had mastadenitis. According to the expression of hormone receptors, breast cancer can be divided into four subtypes: Luminal A breast cancer is hormone-receptor positive (estrogen-receptor and/or progesterone-receptor positive), HER2 negative, and has low levels of the protein Ki-67. Luminal B breast cancer is hormone-receptor positive (estrogen-receptor and/or progesterone-receptor positive), and either HER2 positive or HER2 negative with high levels of Ki-67. HER2-enriched breast cancer is hormone-receptor negative (estrogen-receptor and progesterone-receptor negative) and HER2 positive. Triple-negative/basal-like breast cancer is hormone-receptor negative (estrogen-receptor and progesterone-receptor negative) and HER2 negative.

**Table 1:** Clinicopathological characteristics of the patients in our study.

Variables			Normal		Breast Cancer		Benign	
			Premenopausal (N = 62)	menopausal (N = 70)	Premenopausal (N = 62)	menopausal (N = 70)	Premenopausal (N = 62)	menopausal (N = 70)
**Age, median (range)**			42(25-54)	57(47-79)	41(25-51)	57(47-79)	41(27-51)	57(48-75)
**Family history of breast cancer**			2	3	4	5	2	4
**HRT history**			0	0	0	0	0	0
**menstrual cycle**								
		week1	13	NA	13	NA	13	NA
		Week2	16	NA	16	NA	16	NA
		Week3	15	NA	15	NA	15	NA
		Week4	18	NA	18	NA	18	NA
**T stage**								
		T1	NA	NA	23	25	NA	NA
		T2	NA	NA	31	40	NA	NA
		T3	NA	NA	8	5	NA	NA
**N stage**								
		N0	NA	NA	34	51	NA	NA
		N1	NA	NA	24	16	NA	NA
		N2	NA	NA	4	3	NA	NA
**M stage**								
		M0	NA	NA	62	70	NA	NA
		M1	NA	NA	0	0	NA	NA
**Type**								
		luminal A	NA	NA	5	5	NA	NA
		luminal B	NA	NA	35	39	NA	NA
		HER-2 enriched	NA	NA	19	21	NA	NA
		Triple negative	NA	NA	3	5	NA	NA

NA, not applicable; HER-2, human epidermal growth factor receptor 2; HRT, hormone replacement therapy.

### Comparison of Breast Cancer Group With Normal Control and Benign Group

There was no significant difference in FGT in breast cancer group compared with normal control group and benign lesion group (**Table 2**).

**Table 2:** Comparison of FGT and its AUC among cancer vs control and cancer vs benign.

Menopause status	Cancer vs Control				Cancer vs Benign			
	Cancer(%)	Control(%)	P	AUC	Cancer(%)	Benign(%)	P	AUC
Premenopausal	16.4	17.3	0.689	0.524	16.4	17.0	0.768	0.518
Postmenopausal	9.4	8.2	0.093	0.598	10.7	9.4	0.749	0.482

BPE_I_ was significantly different in breast cancer group compared with the normal control group. The most significant difference occurred in the late enhancement phase in premenopausal women (AUC=0.648) and in the middle enhancement phase in postmenopausal women (AUC=0.618). There was no significant difference in BPE_I_ compared with benign lesions (**Table 3**).

**Table 3:** Comparison of BPE _I_ and its AUC among cancer vs control and cancer vs benign.

Menopause status	Phase	Cancer vs Control				Cancer vs Benign			
		Cancer (%)	Control (%)	P	AUC	Cancer (%)	Benign (%)	P	AUC
Premenopausal	Early	26.50	24.70	0.391	0.551	26.50	26.40	0.985	0.501
	Mid	32.30	29.90	0.043	0.621	32.30	32.00	0.565	0.534
	Late	35.90	32.30	0.013	0.648	35.90	34.80	0.329	0.558
Postmenopausal	Early	25.55	23.10	0.052	0.609	25.55	22.70	0.052	*0.609*
	Mid	27.25	24.70	0.035	0.618	27.25	24.95	0.052	*0.609*
	Late	29.10	26.25	0.114	0.588	29.10	26.55	0.088	0.595

BPE_V_ was higher in the cancer group than in the control and benign groups (P < 0.05). The most significant difference occurred in the middle enhancement phase in premenopausal women (Cancer vs Control AUC=0.715 and Cancer vs Benign AUC= 0.622) and in the early enhancement phase in postmenopausal women (Cancer vs Control AUC=0.684 and Cancer vs Benign AUC= 0.633). (**Table 4**).

**Table 4:** Comparison of BPE_V_ and its AUC among cancer vs control and cancer vs benign.

Menopause status	Phase	Cancer vs Control				Cancer vs Benign			
		Cancer (%)	Control (%)	P	AUC	Cancer (%)	Benign(%)	P	AUC
Premenopausal	Early	30.50	19.50	0.006	0.665	30.50	24.60	0.364	0.554
	Mid	48.10	32.20	0.001	0.715	48.10	38.90	0.041	0.622
	Late	54.30	40.20	0.002	0.687	54.30	48.20	0.173	0.581
Postmenopausal	Early	17.50	13.75	0.003	0.684	17.50	12.85	0.017	0.633
	Mid	26.00	21.70	0.008	0.647	26.00	25.60	0.244	0.565
	Late	29.85	25.20	0.019	0.631	29.85	30.50	0.421	0.545

## Discussion

In the present study, we found that BPE_V_ in the cancer group had increased significantly. Higher BPE_V_ was correlated with an increased risk of breast cancer. The results of previous studies on the association between BPE, FGT and breast cancer were confused by hormone concentrations (12, 20–23), leading to deviations and even opposite conclusions. King et al. (15) reported that as hormone levels decreased after menopause, the mammary glands atrophied and the metabolism declined. In the present study, the patients in the three groups were matched not only for age but also for menopausal status and menstrual cycle. This eliminated the impact of physiological changes on FGT and BPE. Additionally, we only enrolled untreated patients to eliminate the influence of tamoxifen, aromatase inhibitors and hormone replacement therapy.

King (12) compared FGT and BPE of breast cancer before and after menopause with that of the normal control group, and found that BPE of the two groups had significant difference before and after menopause, while FGT showed no significant difference. Our results on FGT are consistent with those of King (12). Studies on BPE demonstrated that BPE_V_ had significant differences in breast cancer compared with normal women and women with benign lesions, with a higher AUC value than BPE_I_. BPE_I_ of breast cancer group is different from that of the normal control group, but not significantly different from the benign lesion group. This may be because benign breast lesions are mostly affected by estrogen and progesterone, and the increased blood supply of breasts. However, the difference of BPE_V_ between breast cancer and benign lesions indicates that the area of breast tissue enhancement in breast cancer patients is more extensive than benign lesions” in the methods. Therefore, it indicated that BPE_V_ has a stronger association with the risk of breast cancer than BPE_I_.

Recently, Wu et al. (23) reported that the wash-in slope variance (WISV), signal enhancement ratio volume (SERV), and BPE% were associated with breast cancer. When the WISV, SERV, and BPE% values were quantitatively assessed in the cancer and benign groups, the AUCs of WISV, SERV, and BPE% were 0.65, 0.63, and 0.64, respectively. These findings regarding BPE_I_ and BPE_V_ in the cancer and benign groups are similar to our results. Their study focused on the differences between benign lesions and breast cancer. However, patients were not matched for menopausal status and menstrual cycle in their study. In addition, BPE_V_ had the maximum AUC between the cancer and benign groups or between the cancer and control groups.

In the studies by King et al. (12) and Wu et al. (23), BPE was only evaluated at one time point: 90 s (k-space center time) after contrast agent administration. Similarly, Dontchos et al. (20) performed their evaluation at 110 s. In the present study, we evaluated BPE_I_ and BPE_V_ at 60, 180, and 300 s after contrast agent administration (k-space center time). And we found that the best phase for assessing the risk of breast cancer was the middle enhancement phase in the premenopausal women and the early enhancement phase in the postmenopausal women.

Quantitative assessment of BPE_V_ is necessary to optimize the treatment strategy in time and improve the therapeutic effect. Gail’s model has been widely used to predict the risk of breast cancer. With addition of the mammary gland density to this model, the prediction accuracy increased with an AUC of 0.602 to 0.670 to and AUC of 0.620 to 0.680 (24–28). In 2013, a meta-analysis of women with high-risk breast cancer who underwent preventive therapy using selective estrogen receptor modulators showed that at the 65-month follow-up, the incidence of breast cancer had decreased to 38% (hazard ratio, 0.62; 95% confidence interval, 0.56–0.69) (29). A major advantage of our study is that BPE_V_ demonstrated a high association of the high risk of breast cancer with an AUC of 0.684 to 0.715. Moreover, BPE_V_ can be used to evaluate the efficacy of preventive therapy after 6 months (30). Therefore, the application of BPE_V_ could further reduce the incidence of breast cancer. Our study compensated for the lack of quantitative measurements in previous research.

This is a retrospective study of 17274 women who underground breast MRI collected for 5 years. However, in these cases, only 132 women with bilateral normal breast met the inclusion criteria, which led to a small sample size of the study. The first limitation of our study is the low time resolution of the MRI dynamic enhanced scans and the relatively low number of scanning phases. The second limitation is that our quantitative software could not distinguish between diseased tissue and normal glands. To avoid lesion-associated effects, we only evaluated the contralateral breast in the benign and cancer groups. Future studies will continue to add the phases of enhanced scanning, improve the time resolution, and shorten the scanning interval. More data regarding the correlation of BPE_I_ and BPE_V_ with the risk of breast cancer are needed. Thirdly, our study only evaluated data regarding breast lesions; further research should be performed with a focus on the glands before lesions develop.

In conclusion, we developed quantitative software which can help assess FGT and BPE. Our study showed that increased BPE_V_ is correlated with a higher risk of breast cancer in both premenopausal and postmenopausal women. BPE_V_ is an important factor which has a high association with the risk of breast cancer by breast MRI.

## Data Availability Statement

The raw data supporting the conclusions of this article will be made available by the authors, without undue reservation.

## Ethics Statement

Ethical review and approval was not required for the study on human participants in accordance with the local legislation and institutional requirements. Written informed consent for participation was not required for this study in accordance with the national legislation and the institutional requirements.

## Author Contributions

All authors contributed to the conception or design of the work, the acquisition, analysis, and interpretation of data for the work, and to the drafting the work or revising it critically for important intellectual content. The authors approved of the final submitted version to be published.

## Funding

This work was supported by the National Natural Science Foundation of China (Grant No. 82071878), Clinical Research Plan of SHDC (SHDC2020CR2008A), Shanghai Anticancer Association FLIGHT PROJECT (SACA-AX-201903), Shanghai Science and Technology Foundation (19DZ1930502) and Shanghai Engineering Research Center of Artificial Intelligence Technology for Tumor Diseases (2020-008).

## Conflict of Interest

The authors declare that the research was conducted in the absence of any commercial or financial relationships that could be construed as a potential conflict of interest.

## Publisher’s Note

All claims expressed in this article are solely those of the authors and do not necessarily represent those of their affiliated organizations, or those of the publisher, the editors and the reviewers. Any product that may be evaluated in this article, or claim that may be made by its manufacturer, is not guaranteed or endorsed by the publisher.
