# Supplementary material for: Fibroglandular Tissue and Background Parenchymal Enhancement on Breast MR Imaging Correlates With Breast Cancer
Source: Front Oncol. 2021 Sep 30;11:616716. doi: 10.3389/fonc.2021.616716 (PMC8515131; doi:10.3389/fonc.2021.616716)
Supplement: Supplementary file 1 [file Table_1.docx]

Supplementary table S1. Scanning parameters of 1.5T breast MRI protocol

| MRI Sequence | TE (msec) | TR (msec) | Section  Thickness (mm) | Field of  View (mm) | Matrix |
| --- | --- | --- | --- | --- | --- |
| Scout | 8.8 | 20.0 | 160 | 360 | 192×64 |
| Axial T2-weighted fat suppressed | 68 | 4008 | 5 | 360 | 320×256 |
| Axial T1-weighted non–fat suppressed | 5.3 | 12.9 | 5 | 360 | 285×256 |
| Axial T1-weighted fat suppressed | 4.8 | 29.0 | 1.5 | 360 | 360×360 |

MRI = magnetic resonance imaging, TE = echo time, TR = repetition time
